# Association of Sex With Risk of 2-Year Revision Among Patients Undergoing Total Hip Arthroplasty

**DOI:** 10.1001/jamanetworkopen.2021.10687

**Published:** 2021-06-02

**Authors:** Amanda Chen, Liz Paxton, Xinyan Zheng, Raquel Peat, Jialin Mao, Alexander Liebeskind, Laura E. Gressler, Danica Marinac-Dabic, Vincent Devlin, Terri Cornelison, Art Sedrakyan

**Affiliations:** 1 Department of Population Health Sciences, Weill Cornell College of Medicine, New York, New York; 2 Kaiser Permanente, San Diego, California; 3 Office of Orthopedic Devices, Office of Product Evaluation and Quality, Center for Devices and Radiological Health, US Food and Drug Administration, Silver Spring, Maryland; 4 Office of Clinical Evidence and Analysis, Center for Devices and Radiological Health, US Food and Drug Administration, Silver Spring, Maryland; 5 Health of Women Program, Center for Devices and Radiological Health, US Food and Drug Administration, Silver Spring, Maryland

## Abstract

**Importance:**

The worldwide population is aging and includes more female individuals than male individuals, with higher rates of total hip arthroplasty (THA) among female individuals. Although research on this topic has been limited to date, several studies are currently under way.

**Objectives:**

To evaluate the association between sex and 2-year revision after THA.

**Design, Setting, and Participants:**

This cohort study used data from statewide databases in New York and California between October 1, 2015, and December 31, 2018. Patients 18 years or older with osteoarthritis who underwent THA and had sex recorded in the database were included in the analysis.

**Exposure:**

Total hip arthroplasty.

**Main Outcomes and Measures:**

The outcome of interest was the difference in early, all-cause revision surgery rates after primary THA between women and men. The association of sex with the revision rate was examined using Cox proportional hazards regression analysis.

**Results:**

Of 132 826 patients included in the study, 74 002 (55.7%) were women; the mean (SD) age was 65.9 (11.0) years, and the median follow-up time was 1.3 years (range, 0.0-3.0 years). The 2-year revision rate was 2.5% (95% CI, 2.4%-2.6%) among women and 2.1% (95% CI, 2.0%-2.2%) among men. After adjusting for demographic characteristics, comorbidities, and facility volume, a minimal clinically meaningful difference was observed in revision rates despite women having a higher risk of all-cause revision compared with men (hazard ratio, 1.16; 95% CI, 1.07-1.26; *P* < .001). The risk of revision was increased among women compared with men in the subgroup of patients who were younger than 55 years (hazard ratio, 1.47; 95% CI, 1.20-1.81; *P* < .001).

**Conclusions and Relevance:**

In this cohort study, no clinically meaningful difference in all-cause revision rates after primary THA was found between men and women at 2-year follow-up. The modest difference in the risk of revision between men and women in a small subgroup of patients younger than 55 years suggests that the risk of revision in this population should be studied further.

## Key Points

**Question:**

Is there an association between sex and the 2-year revision rate after total hip arthroplasty?

**Findings:**

In this cohort study of 132 826 patients with osteoarthritis, no clinically meaningful difference was found in all-cause rates of revision between men and women. The absolute difference in overall revision risk among men and women was small at 1- and 2-year follow-up.

**Meaning:**

The findings suggest that sex is not associated with the risk of 2-year revision after total hip arthroplasty.

## Teaser

This cohort study evaluates the association between sex and 2-year revision after total hip arthroplasty as well as the association between sex and risk of revision.

## Introduction

Total hip arthroplasty (THA) is a common and effective elective procedure for the treatment of end-stage osteoarthritis, a leading cause of disability.^1^ More than 2.5 million people in the US have received a total hip replacement,^2^ and the number of primary THA procedures conducted annually is projected to increase by 71% over the next 10 years.^3^ Although THA is associated with improved patient health-related quality of life,^4,5^ some implants may fail, leading to the need for higher-risk revision procedures. These procedures are associated with increased risk of complications or mortality.^6^ Each year, more than 3% of all arthroplasties performed are hip revision procedures,^7^ with the mean cost of these procedures exceeding $77 000.^3^

With an aging population that includes more female individuals than male individuals, it is important to understand whether revision surgery is performed more often among female individuals. A number of studies have documented higher rates of THA among female individuals.^2,8,9^ Several factors might contribute to the possible disparity in the use of revision procedures. Compared with male patients, female patients are more likely to have osteoarthritis,^10,11^ worse functional status,^10,12,13,14^ and a more advanced stage of disease with greater disability at the time of surgery.^10,12,13,14^ However, the importance of sex as a risk factor for revision surgery is unclear. Some studies suggest an increased risk of revision associated with the male sex,^15,16,17^ whereas others suggest an increased risk associated with the female sex^18,19,20,21^ or no difference in risk between male and female patients.^22,23^ These differences among studies may be attributed to numerous factors, such as differences in the size or demographic characteristics of the cohort, implant characteristics, the length of follow-up, and the definition of revision surgery.

The main objective of this study was to use the most recent available data from 2 states with large populations (New York and California) to examine the differences in early revision surgery rates after primary THA between women and men. The secondary objective was to identify modifiers for the association between sex and all-cause revision.

## Methods

### Data Source

In this cohort study, data obtained from the New York State Department of Health Statewide Planning and Research Cooperative System (SPARCS) and the California Office of Statewide Health Planning and Development (OSHPD) were analyzed. SPARCS collects demographic and clinical information for every patient discharged from nonfederal acute care facilities in New York, including inpatient and outpatient surgery services, ambulatory surgery centers, and emergency departments. The OSHPD maintains abstracts of inpatient discharge, emergency, and ambulatory surgery encounters in California-licensed health care facilities. The institutional review board of Weill Cornell College of Medicine approved this study and waived the requirement for informed consent from participants because data were deidentified. This study followed the Strengthening the Reporting of Observational Studies in Epidemiology (STROBE) reporting guideline.^24^

We performed a data quality check of the OSHPD by comparing the estimated revision rates of THA in Kaiser Permanente hospitals with those reported by Kaiser Registry partners. The estimates were similar. Unique encrypted identifiers for patients were available in both data sets, allowing for longitudinal follow-up in each cohort. Using *International Statistical Classification of Diseases, Tenth Revision, Clinical Modification (ICD-10-CM)* and *International Statistical Classification of Diseases, Tenth Revision, Procedure Coding System* (*ICD-10-PCS*) codes, we identified patients who had an osteoarthritis diagnosis and underwent a total hip replacement from October 1, 2015, to December 31, 2017, in California and from January 1, 2016, to December 31, 2018, in New York State (eTables 1-3 in the Supplement). To restrict procedures to primary THA, we excluded patients who underwent a concurrent hip implant removal or revision or concurrent hip resurfacing during the index procedure admission. In addition, we excluded patients who were not residents of New York or California, patients who were younger than 18 years, and patients whose sex was missing from the databases at the time of the index procedure admission (eFigure in the Supplement). Observations in which data were missing were as follows: for sex, fewer than 11 (<1%); for race/ethnicity, 756 (<1%); for insurance variables, 35 (<1%); thus, listwise deletion was performed.

The following patient characteristics were assessed at the time of the index procedure admission: age (<55, 55-64, or ≥65 years), sex (male or female), race/ethnicity (White, Black, Hispanic, or other [American Indian or Alaska Native, Asian, or Native Hawaiian or other Pacific Islander]), insurance type (Medicare, Medicaid, commercial, self-pay, or other), state (New York or California), and comorbidities (morbid obesity, hypertension, chronic obstructive pulmonary disease, congestive heart failure, diabetes, depression, peripheral vascular disease, coagulopathy, hypothyroidism, valvular disease, kidney disease, cancer, and hypercholesterolemia) (eTables 1-3 in the Supplement). Race/ethnicity categories provided in the SPARCS and OSHPD data sets were aggregated to create 4 categories to assess variation in the outcome measures by race/ethnicity. We used mean annual volume of THAs as a facility-level characteristic. The volume was calculated by summing the annual number of primary THA procedures performed for the most recent 5 years for each state (New York, 2014-2018; California, 2013-2017) and then calculating mean values for years in which at least 1 procedure was performed. Facility volume was treated as a continuous variable in the main analysis and categorized as low (≤40th percentile), medium (>40th percentile to ≤80th percentile), and high (>80th percentile) in the stratified analysis using the 40th percentile and 80th percentile as reported in a previous study.^25^

The outcome of interest was all-cause revision, defined as the addition, removal, or replacement of a THA implant (eTables 1-3 in the Supplement). Using *ICD-10-CM* and *ICD-10-PCS* procedure codes, we identified revision events that occurred on the same side of the hip as the index replacement procedure.

### Statistical Analysis

Baseline characteristics of patients were summarized using counts and percentages. The facility mean annual volume was summarized using median values and interquartile ranges. Because of the large sample size, the baseline differences between men and women were compared using standardized mean differences (SMDs) calculated according to the method proposed by Cohen.^26^ The SMD associated with facility volume was based on rank statistics because of the right-skewed distribution. In the main analysis, we calculated the cumulative incidence of revision by sex using Kaplan-Meier analysis. Patients were censored at the time of revision or death or at the end of the study, whichever occurred first. The association of sex with the revision rate was then examined using a Cox proportional hazards regression model with a robust sandwich estimator to account for facility clusters. We used 3 nested Cox proportional hazards regression models. The first model included sex as the sole explanatory variable. The second model adjusted for age, race/ethnicity, insurance status, and facility mean annual volume. In the third model, comorbidities were additionally adjusted. These adjustments for confounding were made to address potential sources of bias.

The following variables were evaluated for effect modifiers of the association between sex and all-cause revision: age, race/ethnicity, insurance, and facility volume. We tested the 2-way interaction between sex and each variable in a fully adjusted model. Using these models with interaction terms, we examined the rate of revision for women and men within each subgroup. Because all of the models showed some degree of interaction, we then performed analyses stratified by each variable. We repeated the fully adjusted Cox proportional hazards regression analysis with a robust sandwich estimator within each stratum to compare the difference in revision rates between men and women. *P* values were 2-sided, and *P* < .05 was considered statistically significant. All analyses were performed using SAS, version 9.4 (SAS Institute Inc).

## Results

In this cohort, 78 149 patients in New York and 77 426 in California underwent primary THA. After exclusion criteria were applied, 65 109 patients in New York (49.0%) and 67 717 patients in California (51.0%) were included in the final analytical cohort (eFigure in the Supplement). The cohort included 74 002 (55.7%) women, and the mean (SD) age was 65.9 (11.0) years; the mean (SD) age of women was 67.1 (10.9) years, and the mean (SD) age of men was 64.2 (10.9) years (SMD, 0.26; 95% CI, 0.25-0.27). Most patients were 65 years or older (74 174 [55.8%]), White (106 185 [79.9%]), and Medicare beneficiaries (67 223 [50.6%]). Among patients with comorbid conditions, 15 053 women (20.3%) had hypothyroidism compared with 3815 men (6.5%) (SMD, 0.42; 95% CI, 0.40-0.43), and 10 534 women (14.2%) had depression compared with 4080 men (6.9%) (SMD, 0.24; 95% CI, 0.23-0.25) (Table).

**Table.** Patient Characteristics

Characteristic	Patients, No. (%)			SMD (95% CI)
	Total (N = 132 826)	Male (n = 58 824)	Female (n = 74 002)	
Age, mean (SD), y	65.9 (11.0)	64.2 (10.9)	67.1 (10.9)	NA
Age, y				
<55	18 811 (14.2)	10 087 (17.1)	8724 (11.8)	0.26 (0.25 to 0.27)
55-64	39 841 (30.0)	19 977 (34.0)	19 864 (26.8)	
≥65	74 174 (55.8)	28 760 (48.9)	45 414 (61.4)	
Race/ethnicity^a^				
White	106 185 (79.9)	47 116 (80.1)	59 069 (79.8)	0.03 (0.02 to 0.04)
Black	8778 (6.6)	3878 (6.6)	4900 (6.6)	
Hispanic	9373 (7.1)	4243 (7.2)	5130 (6.9)	
Other^b^	7734 (5.8)	3216 (5.5)	4518 (6.1)	
Insurance				
Medicare^a^	67 223 (50.6)	25 759 (43.8)	41 464 (56.0)	0.27 (0.25 to 0.28)
Medicaid	6345 (4.8)	3086 (5.2)	3259 (4.4)	
Commercial	55 698 (41.9)	27 721 (47.1)	27 977 (37.8)	
Self-pay	693 (0.5)	356 (0.6)	337 (0.5)	
Other	2832 (2.1)	1886 (3.2)	946 (1.3)	
State				
New York	65 109 (49.0)	29 007 (49.3)	36 102 (48.8)	−0.01 (−0.02 to 0.00)
California	67 717 (51.0)	29 817 (50.7)	37 900 (51.2)	
Comorbidity^c^				
Morbid obesity	7654 (5.8)	3136 (5.3)	4518 (6.1)	0.03 (0.02 to 0.04)
Hypertension	74 331 (56.0)	34 409 (58.5)	39 922 (53.9)	−0.09 (−0.10 to −0.08)
COPD	8102 (6.1)	3300 (5.6)	4802 (6.5)	0.04 (0.03 to 0.05)
Congestive heart failure	2933 (2.2)	1555 (2.6)	1378 (1.9)	−0.05 (−0.06 to −0.04)
Diabetes	17 940 (13.5)	8964 (15.2)	8976 (12.1)	−0.09 (−0.10 to −0.08)
Depression	14 614 (11.0)	4080 (6.9)	10 534 (14.2)	0.24 (0.23 to 0.25)
Peripheral vascular disease	5473 (4.1)	2386 (4.1)	3087 (4.2)	0.006 (−0.005 to 0.02)
Coagulopathy	3181 (2.4)	1602 (2.7)	1579 (2.1)	−0.04 (−0.05 to −0.03)
Hypothyroidism	18 868 (14.2)	3815 (6.5)	15 053 (20.3)	0.42 (0.40 to 0.43)
Valvular disease	5313 (4.0)	2236 (3.8)	3077 (4.2)	0.02 (0.007 to 0.03)
Kidney disease	7124 (5.4)	3420 (5.8)	3704 (5.0)	−0.04 (−0.05 to −0.03)
Cancer	1478 (1.1)	872 (1.5)	606 (0.8)	−0.06 (−0.07 to −0.05)
Hypercholesterolemia	4115 (3.1)	1955 (3.3)	2160 (2.9)	−0.02 (−0.03 to −0.01)
Annual mean facility volume, median (IQR), No. of THAs^d^	266.8 (145.2-508.8)	266.8 (145.2-508.8)	266.8 (145.7-508.8)	0.01 (0.001 to 0.02)

Abbreviations: COPD, chronic obstructive pulmonary disease; IQR, interquartile range; SMD, standardized mean difference; THA, total hip arthroplasty.

^a^ Overall, 756 patients (0.57%) had missing race/ethnicity, and 35 (0.03%) had missing insurance.

^b^ Other included American Indian or Alaska Native, Asian, and Native Hawaiian or other Pacific Islander.

^c^ The *International Statistical Classification of Diseases, Tenth Revision* diagnosis codes used to identify comorbidities are listed in eTable 1 in the Supplement.

^d^ Data are from 2014 to 2018 in New York and from 2013 to 2017 in California; the SMD was based on rank statistics.

**Figure 1.**
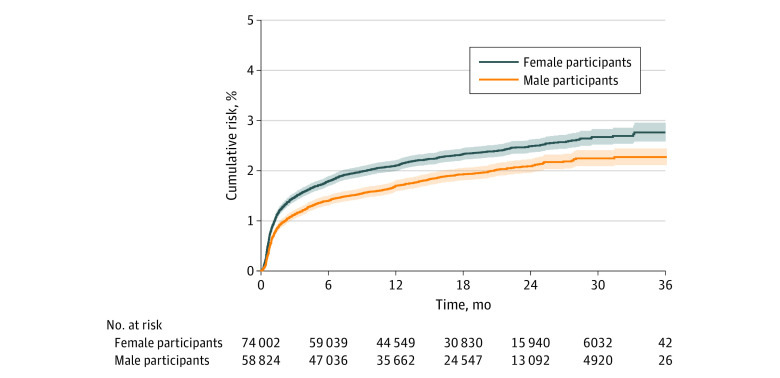
Kaplan-Meier Analysis of All-Cause Revision Among Patients Undergoing Total Hip Arthroplasty by Sex Shaded areas indicate 95% CIs.

The all-cause revision rate was higher among women than among men according to the Kaplan-Meier analysis (Figure 1). The 2-year revision rate was 2.5% (95% CI, 2.4%-2.6%) among women and 2.1% (95% CI, 2.0%-2.2%) among men (log-rank test: *P* < .001). In the unadjusted Cox proportional hazards regression model, the risk of all-cause revision was 22% higher among women compared with men (hazard ratio [HR], 1.22; 95% CI, 1.13-1.33; *P* < .001) (eTable 4 in the Supplement) . After adjusting for demographic characteristics and facility mean annual volume, the risk of revision was 20% higher among women compared with men (HR, 1.20; 95% CI, 1.11-1.31; *P* < .001). When comorbidities were added to the adjusted model, women had a 16% higher risk of revision (HR, 1.16; 95% CI, 1.07-1.26; *P* < .001). For revisions performed because of sepsis, the risk of revision was 12% lower among women compared with men in the unadjusted Cox proportional hazards regression model (HR, 0.78; 95% CI, 0.67-0.90; *P* < .001) and 15% lower among women after adjustment (HR, 0.75; 95% CI, 0.64-0.87; *P* < .001)

**Figure 2.**
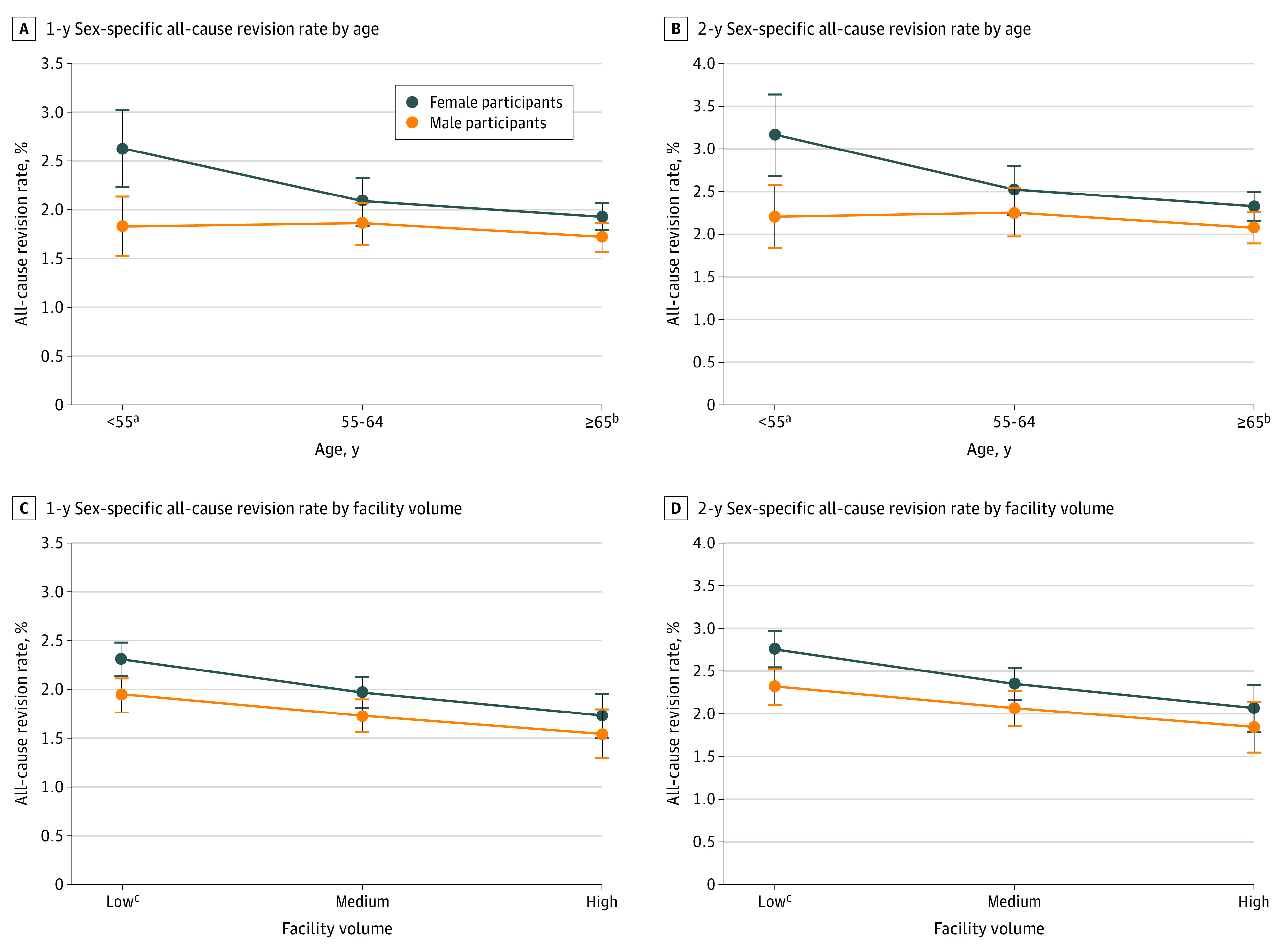
One-Year and 2-Year Estimated Sex-Specific All-Cause Revision Rates by Age and Facility Volume in the Fully Adjusted Model *P* = .06 for the overall interaction with age, and *P* = .14 for the overall interaction with facility volume. ^a^*P* < .001. ^b^*P* = .04. ^c^*P* = .004.

**Figure 3.**
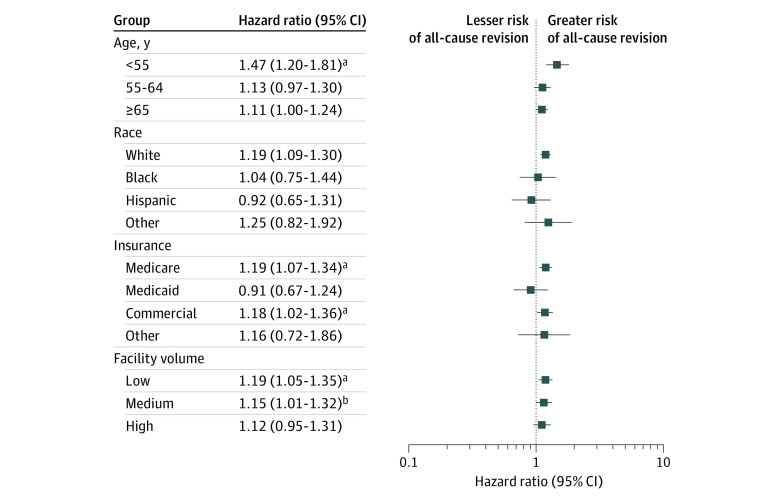
Comparison of Risk of All-Cause Revision by Sex in the Stratified Analysis Markers indicate hazard ratios, with horizontal lines representing 95% CIs. ^a^*P* < .001. ^b^*P* < .05.

Revision rates differed significantly among women and men in the interaction analysis at both 1 and 2 years (Figure 2). The stratified analysis showed that women had a higher risk for revision than men if they were younger than 55 years (HR, 1.47; 95% CI, 1.20-1.81; *P* < .001), were White (HR, 1.19; 95% CI, 1.09-1.30; *P* < .001), had Medicare (HR, 1.19; 95% CI, 1.07-1.34; *P* < .001) or commercial insurance (HR, 1.18; 95% CI, 1.02-1.36; *P* < .001), or had the index procedure performed at a low-volume facility (HR, 1.19; 95% CI, 1.05-1.35; *P* < .001) (Figure 3). There were no differences in revision rates among patients who were Black or Hispanic, those who had Medicaid insurance, or those who had the index procedure performed at high-volume facilities.

## Discussion

In this cohort study of 132 286 patients who underwent primary THA in 2 US states with large populations, after adjustment for demographic, clinical, and facility-level factors, no clinically meaningful difference was observed in the risk of revision between men and women. Although women had a slightly higher risk of revision compared with men overall, both men and women had a low baseline revision risk, and the absolute difference was minimized with tighter covariate adjustment in our models. Although the risk of revision owing to infection was lower for women compared with men, our estimate was consistent with that reported in a prior study,^27^ and the difference in overall revision rates may have been associated with aseptic causes of revision.

In a previous study^19^ in which Kaiser Permanente registry data were used, a slightly lower estimated risk was found compared with the results in the present study. The findings from that previous investigation included high treatment failure rates among women who received implants with metal-on-metal bearing surfaces. The inclusion of patients receiving metal-on-metal implants (which are now no longer widely used), who represented 13.8% of the study cohort, may have contributed to the more pronounced difference in all-cause revision rates between men and women.^19^ We examined a larger and more diverse cohort in this study and captured revisions occurring at any hospital within New York and California after the initial THA that may not have been reported to registries. The findings of the present study using contemporaneous data support the safety of THA in the post–metal-on-metal implant era for men and women overall.

Our findings are similar to results from a study that used older, international data from a Scandinavian registry^28^ and to those of a single-center series^23^ that found no difference in the revision rates between men and women. The single-center series^23^ also revealed reasons for undergoing revision surgery that were similar to those found in our study, such as aseptic loosening, implant failure, periprosthetic fracture, polyethylene wear, osteolysis, and infection. A US study from 2010^22^ that used Medicare claims data for patients older than 65 years revealed no difference in the risk of revision between men and women.^22^ Prior research has also shown that the use of a larger femoral head size, which is likely more common in women, is associated with a lower risk of revision^29^ and may explain why sex was not associated with a clinically meaningful difference in revision rates in our study.

In addition, we investigated whether the risk of revision differed by sex in specific subgroups. Previous studies reported that younger patient age was associated with an increased risk of revision in both the short^22,27^ and long term.^15,16,17,30^ However, some studies^15,22,31^ were limited to individuals eligible for Medicare even though more than one-third of patients undergoing THA in the US are not represented by the Medicare population.^15^ This finding was supported by our study because only 50.6% of the study sample was insured by Medicare. Despite some studies focusing on risk of revision among older adults,^22,32^ other research has shown that the number of revisions among patients aged 45 and 64 years has increased by 42% from 2007 to 2013.^33^ Based on these findings, younger patients may have the greatest risk of revision in the long term. In the present study, compared with the all-cause revision rate among men younger than 55 years, the rate among women in this age group was increased at both 1 and 2 years. Our findings suggest that further study may be warranted as the number of young patients undergoing THA continues to increase.

Some of the differences in outcomes between men and women may have been associated with anatomic differences in morphologic features of the hip, such as the location of the femoral head center or the shape and size of the femoral canal.^23^ In addition, the incidence of early fracture and subsequent revision owing to the use of uncemented devices in women with potentially lower bone quality may contribute to the difference in revision rates between men and women.^34,35^ A study^32^ using data from several major joint replacement registries found a higher risk of revision associated with uncemented THA, particularly among those older than 75 years. In addition, prior research from the Norwegian Arthroplasty Register^36^ showed that the use of uncemented stems was associated with increased risk of revision owing to periprosthetic fracture and dislocation in women older than 55 years. Because the volume of THA procedures is projected to increase, especially among women, further research should be dedicated to addressing disparities in early revision, which may be associated with improved overall outcomes and cost savings.

### Strengths and Limitations

This study has strengths. We were able to capture information about the surgically treated side (laterality) through the use of detailed *ICD-10-CM* and *ICD-10-PCS* codes. Also, the robust sample size allowed for more generalized conclusions about the association of sex with revision rates in the US population.

This study also has limitations. First, the coding system used limited the clinical significance of our findings because it can be subject to errors and did not allow us to exclude THA procedures in which large-head or metal-on-metal hip replacements were used. Second, we were not able to adjust for differences in specific implant designs or account for the effect of femoral head size; prior research^19^ has suggested an association between use of smaller femoral head sizes and a higher risk of revision in women compared with men. Third, we were not able to assess differences in the underlying condition necessitating the surgery or the reason for revision, such as aseptic loosening. Fourth, we were also unable to account for surgical approach; a prior study^37^ showed that the direct anterior approach was associated with lower rates of dislocation and revision because of instability, 1 of the main contributors to early implant failure. Fifth, the timing of our study was limited to short-term follow-up because *ICD-10-CM* and *ICD-10-PCS* codes were only available from October 2015 onward. *International Classification of Diseases, Ninth Revision* procedure codes used before that time did not distinguish laterality.

## Conclusions

In this cohort study, there was no clinically significant difference in the risk of all-cause revision between men and women at 2-year follow-up, even after adjusting for demographic, clinical, and facility-level characteristics. Although the differences in the general patient population were too small to conclude a significant association, we found a modest difference in the risk of revision in a small subgroup of women younger than 55 years compared with men in the same age group. Given the increasing number of younger people undergoing THA, future research should examine the factors associated with differences in the risk of revision by sex in a larger sample of younger patients with longer-term follow-up.

## Notes

Supplement. **eTable 1.** Clinical Codes Used for Conditions **eTable 2.** ICD-10 Diagnosis Codes Used for Malignancy **eTable 3.** Clinical Codes Used for Procedures **eTable 4.** Hazard Ratio of Revision Events Comparing Female to Male in Main Analysis **eFigure.** Flow Chart for Patient Selection
