# Supplementary material for: Association of Sex With Risk of 2-Year Revision Among Patients Undergoing Total Hip Arthroplasty
Source: JAMA Netw Open. 2021 Jun 2;4(6):e2110687. doi: 10.1001/jamanetworkopen.2021.10687 (PMC8173374; doi:10.1001/jamanetworkopen.2021.10687)
Supplement: Supplement. — eTable 1. Clinical Codes Used for Conditions eTable 2. ICD-10 Diagnosis Codes Used for Malignancy eTable 3. Clinical Codes Used for Procedures eTable 4. Hazard Ratio of Revision Events Comparing Female to Male in Main Analysis eFigure. Flow Chart for Patient Selection [file jamanetwopen-e2110687-s001.pdf]

Supplementary Online Content

Chen A, Paxton L, Zheng X, et al. Association of sex with risk of 2-year revision among patients undergoing total hip arthroplasty. *JAMA Netw Open*. 2021;4(6):e2110687. doi:10.1001/jamanetworkopen.2021.10687

eTable 1. Clinical Codes Used for Conditions
eTable 2. ICD-10 Diagnosis Codes Used for Malignancy
eTable 3. Clinical Codes Used for Procedures
eTable 4. Hazard Ratio of Revision Events Comparing Female to Male in Main Analysis
eFigure. Flow Chart for Patient Selection

This supplementary material has been provided by the authors to give readers additional information about their work.

© 2021 Chen A et al. *JAMA Network Open*.

eTable 1. Clinical codes used for conditions	
	**ICD 10 diagnosis codes**
Osteoarthritis	M15-M19
Morbid Obesity	E66.01, E66.2, Z684
Hypertension	I10, I11, I12, I13
COPD	J41-J44
Congestive Heart Failure	I0981, I50
Diabetes	E08, E09, E10, E11, E13
Depression	F320, F321, F322, F323, F324, F325, F3282, F329, F33, F341, F4321
Peripheral vascular disease	I70, I71, I72, I73, I742, I743, I744, I76, I771, I777, I79, K551, K558, K559, Z9582
Coagulopathy	D65-D68.x, D69.1, D69.3-D69.6
Hypothyroidism	E00x, E018, E02, E030, E031, E032, E033, E038, E039
Valvular disease	A52.03, I05.x - I08.x, I09.1, I09.89, I34.x - I39.x, Q23.0 - Q23.3, Z95.2 - Z95.4
Renal disease	N18.3, N18.4, N18.5, N18.6, N18.9, N19.x, Z49.01, Z49.02, Z49.31, Z49.32, Z91.15, Z94.0 - Z94.0, Z99.2
History of cancer/solid tumor	See eTable 2
Hypercholesterolemia	E78.0

© 2021 Chen A et al. *JAMA Network Open*.

eTable 2. ICD 10 diagnosis codes used for malignancy
C000, C001, C002, C003, C004, C005, C006, C008,C01, C020, C021, C022, C023, C028, C024, C029, C07, C080, C081, C089, C030, C031, C039,C040, C041, C048, C049, C060, C061, C050, C051, C052, C059, C062, C0689, C069, C099, C090, C091, C100, C101, C108, C102, C103, C104, C108, C109, C110, C111,C112, C113, C118, C119, C130, C12, C131, C132, C138, C139, C140, C142, C148,C153,C154, C155, C153, C154, C158, C159, C160, C164, C163, C161, C162, C165,C166, C168, C169, C170, C171, C172, C173, C178, C179, C183, C184, C186, C187,C180, C181, C182, C185, C188, C189, C19, C20, C211, C210, C218, C220, C222, C227, C228, C221, C229, C23, C240, C241, C248, C249, C23, C240, C241, C248, C249, C250, C251, C252, C253, C254, C257, C258, C259, C480, C481, C488, C482, C260, C261,C269, C300, C301, C310,C311, C312, C313, C318, C319, C320, C321, C322, C323, C328, C329, C33, C3400, C3410, C342, C3430, C3480, C3490, C384, C37, C380, C381, C382, C388, C383, C390, C399, C410, C411, C412, C413, C4000,C4010, C414, C4020,C4030, C419, C490, C4910, C4920, C493, C494, C495, C496, C478, C498, C499,C430,D030,C4310, D0310, D0311, D0312, C4320, D0320, D0321, D0322, C4330, C4331, C4339, D0330, D0339, C434, D034, C4359, D0351, D0352, D0359, C4360, D0360, D0361, D0362, C4370, D0370, D0371, D0372, C438, D038, C439, D039, C4400, C4401, C4402, C4409, C44101, C44111, C44121, C44191, C44201, C44211, C44221, C44291, C44300, C44301, C44309, C44310, C44311, C44319, C44320, C44321, C44329, C44390, C44391, C44399, C4440, C4441, C4442, C4449, C44500, C44501, C44509, C44510, C44511, C44519, C44520, C44521, C44529, C44590, C44591, C44599, C44601, C44611, C44621, C44691, C44701, C44711, C44721, C44791, C4480, C4481, C4482, C4489, C4490, C4491, C4492, C4499, C50019, C50119, C50219, C50319, C50419, C50519, C50619, C50819, C50919, C50029, C50929, C460, C461, C462, C464, C4650, C463, C467, C469, C55, C530, C531, C538, C539, C58, C541, C542, C543, C549, C540, C548, C569, C5700, C5710, C573, C5720, C574, C52, C510, C511, C512, C519, C577, C578, C579, C61, C6200, C6210,C6290, C600, C601, C602, C609, C6300, C6310, C632, C608, C637, C638, C639, C670, C671, C672, C673, C674, C675, C676, C677, C678, C679, C649, C659,C669, C680, C681,C688, C689, C6940, C6960, C6950, C6900, C6910, C6920, C6930, C6950, C6980, C6990, C710, C711,C712, C713, C714, C715, C716, C717, C718, C719, C7250, C700, C709,C720,C721, C701, C729, C73, C7490, C750, C751, C752, C753, C754, C755, C758, C759, C760,C761, C762, C763, C7640, C7650, C768

© 2021 Chen A et al. *JAMA Network Open*.

**eTable 3. Clinical codes used for procedures**		
	ICD 10 procedure codes	
	Right	Left
Total Hip Replacement	0SR9x	0SRBx
Hip resurfacing	0SU9/A/R end with 0BZ	0SUB/E/S end with 0BZ
**Revision**		
Revision Of Hip Replacement, Not Otherwise Specified	0SW90JZ, 0SWA0JZ, 0SWR0JZ	0SWB0JZ, 0SWE0JZ, 0SWS0JZ
Revision Of Hip Replacement, Both Acetabular And Femoral Components	Removal code 0SP9 + Replacement code 0SR9x	Removal code 0SPB + Replacement code 0SRBx
Revision Of Hip Replacement, Acetabular Component	Removal code 0SP9/A + Replacement code 0SRAx	Removal code 0SPB/E + Replacement code 0SREx
Revision Of Hip Replacement, Femoral Component	Removal code 0SP9/R + Replacement code 0SRRx	Removal code 0SPB/S + Replacement code 0SRSx
Revision Of Hip Replacement, Acetabular Liner And/Or Femoral Head Only	Removal code 0SP9 + Supplement code 0SU9/A/Rx	Removal code 0SPB + Supplement code 0SUB/E/Sx
**Removal**		
Removal of Spacer from Hip Joint	0SP908Z	0SPB08Z
Removal of Liner from Hip Joint	0SP909Z	0SPB09Z
Removal of Resurfacing Device from Hip Joint	0SP90BZ	0SPB0BZ
Removal of Synthetic Substitute from Hip Joint	0SP90JZ	0SPB0JZ
Removal of Synthetic Substitute from Hip Joint, Acetabular Surface	0SPA0JZ	0SPE0JZ
Removal of Synthetic Substitute from Hip Joint, Femoral Surface	0SPR0JZ	0SPS0JZ

© 2021 Chen A et al. *JAMA Network Open*.

eTable 4. Hazard ratio of revision events comparing female to male in main analysis

	Unadjusted	Adjusted Model 1: demographic + facility volume	Adjusted Model 2: demographic + facility volume + comorbidities
Main analysis: All cause revision			
Female	1.22(1.13,1.33)††	1.20(1.11, 1.31)††	1.16(1.07, 1.26) ††
Main analysis: Septic revision			
Female	0.78(0.67,0.90)††	0.77(0.67,0.90)††	0.75(0.64,0.87)††
Male	Ref	Ref	Ref
Subgroup analysis: Age <55, all-cause revision			
Female	1.48(1.21, 1.82)††	1.49(1.22, 1.84)††	1.47(1.20, 1.81)††
Male	Ref	Ref	Ref
Subgroup analysis: low volume centers, all-cause revision			
Female	1.26(1.12, 1.41)††	1.25(1.11, 1.40)††	1.19(1.05, 1.35)††
Male	Ref	Ref	Ref
Cox regression models were used, with robust sandwich estimator accounted for facility cluster. Facility mean annual volume was treated as a continuous variable. * < 0.05 † <0.01 †† < 0.001.			

© 2021 Chen A et al. *JAMA Network Open*.

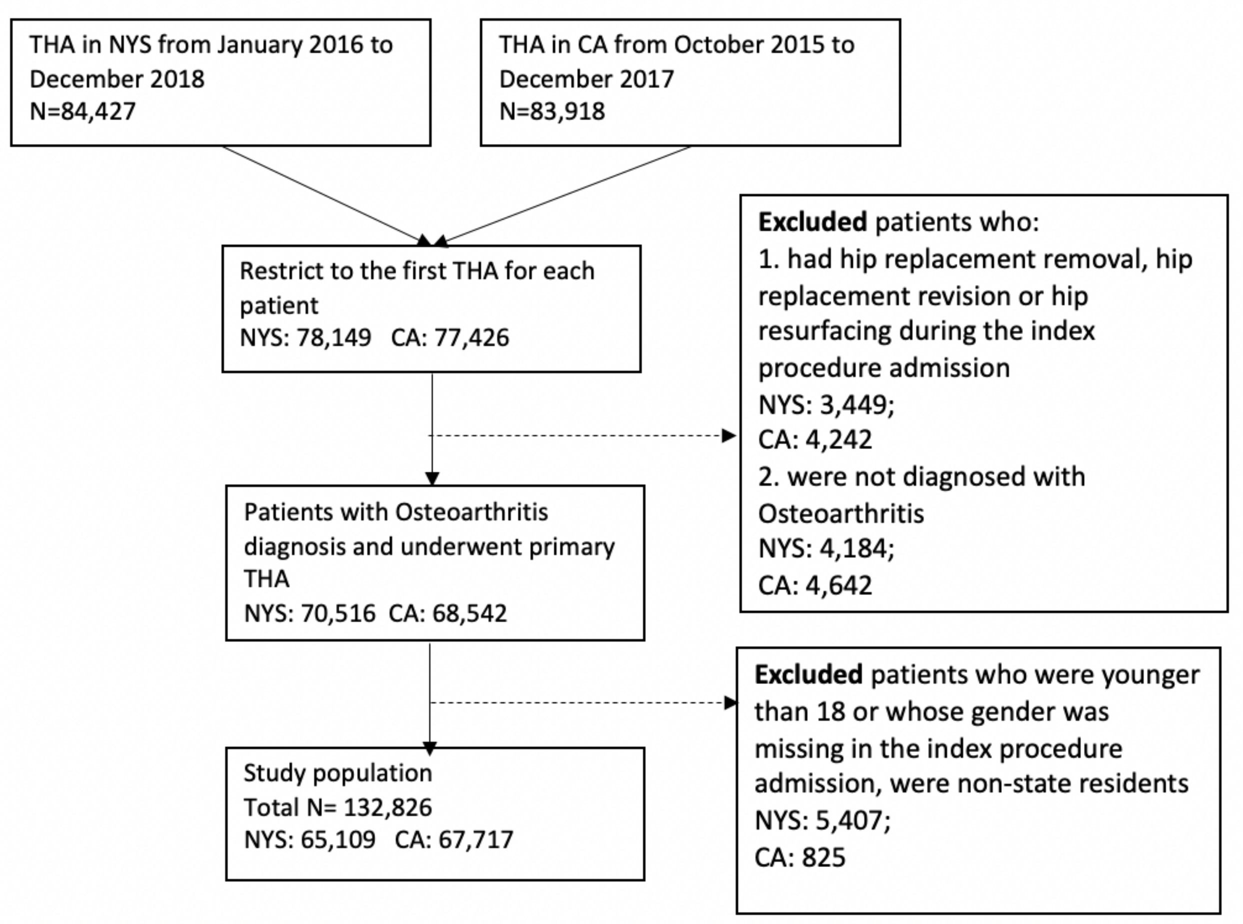

eFigure. Flow chart for patient selection

© 2021 Chen A et al. *JAMA Network Open*.
